# Supplementary material for: Potential Beneficial Role of Nitric Oxide in SARS-CoV-2 Infection: Beyond Spike-Binding Inhibition
Source: Antioxidants (Basel). 2024 Oct 26;13(11):1301. doi: 10.3390/antiox13111301 (PMC11591382; doi:10.3390/antiox13111301)
Supplement: Supplementary file 1 [file antioxidants-13-01301-s001.zip › antioxidants-3258167-supplementary.pdf]

Potential beneficial role of nitric oxide in SARS-CoV-2 infection: beyond spike binding inhibition

Sergio Sánchez-García [1,*], Antonio Castrillo [1,2], Lisardo Boscá [1,2,3,*], Patricia Prieto [3,4,*]

[1]Instituto de Investigaciones Biomédicas Sols-Morreale, CSIC-UAM, Arturo Duperier 4. 28029 Madrid, Spain

[2]Unidad de Biomedicina (Unidad Asociada al CSIC) de la Universidad de Las Palmas de Gran Canaria, Las Palmas, Spain

[3]Centro de Investigación Biomédica en Red de Enfermedades Cardiovasculares (CIBERCV), Av. Monforte de Lemos 3-5, P-11. 28029 Madrid, Spain

[4]Departamento de Farmacología, Farmacognosia y Botánica, Facultad de Farmacia, Universidad Complutense de Madrid, Plaza Ramón y Cajal. 28040 Madrid, Spain

*Correspondence: S.S-G. sgarcia@iib.uam.es; L.B. lbosca@iib.uam.es; P.P. pprieto@ucm.es

Lisardo Boscá; lbosca@iib.uam.es

Instituto de Investigaciones Biomédicas Sols-Morreale,

Arturo Duperier, 4; E-28029 Madrid; Spain

Sergio Sánchez-García; sgarcia@iib.uam.es

Patricia Prieto; pprieto@ucm.es

Facultad de Farmacia UCM

Plaza Ramón y Cajal. 28040 Madrid, Spain

Supplementary material

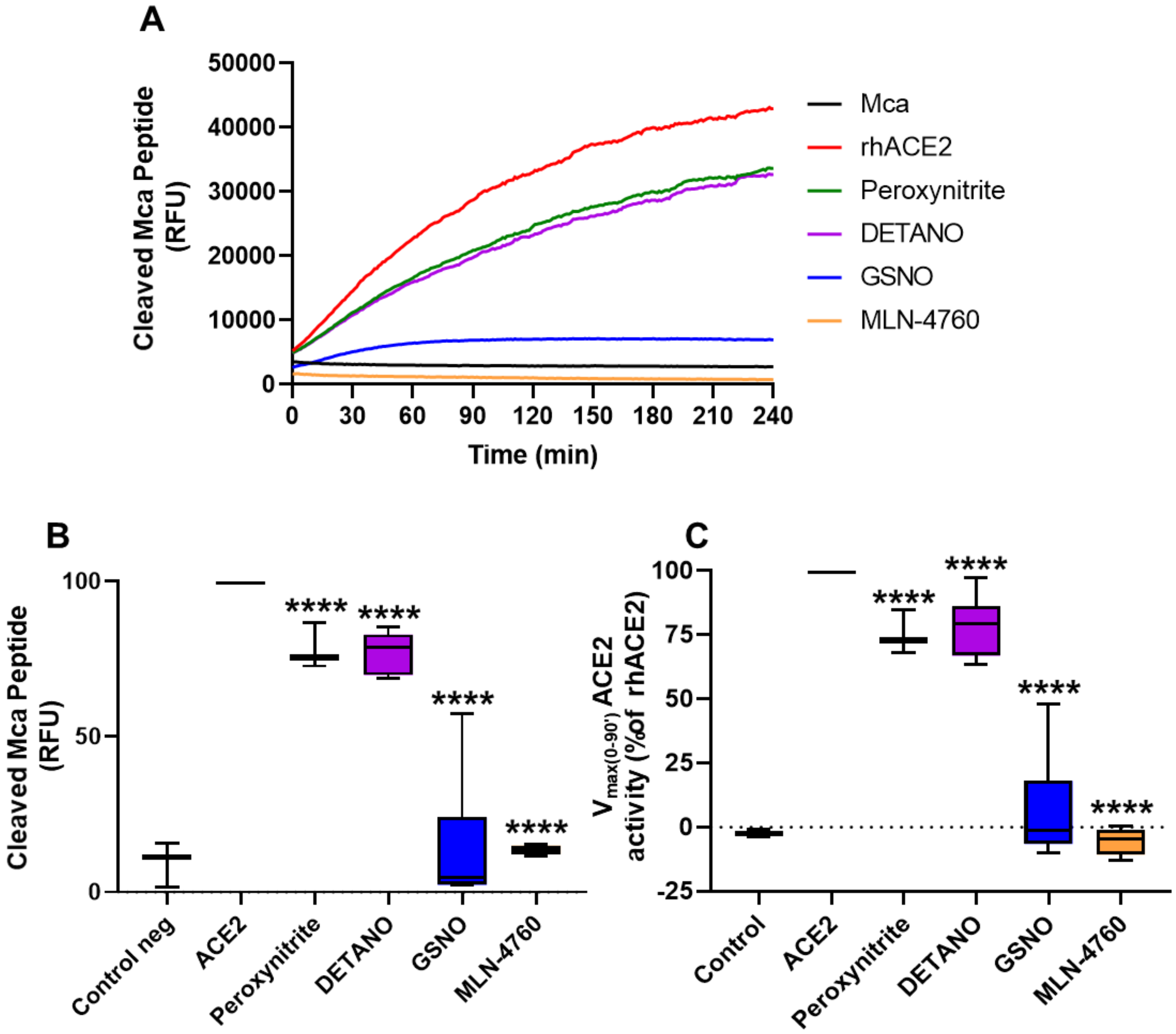

Supplementary figure S1. NO donors and peroxynitrite inhibit the activity of recombinant human ACE2 (extended). (A) ACE2 activity was determined using 10 ng of the recombinant human ACE2 enzyme (rhACE2, 100 ng/ml) and 10 µM of the Mca-ACE2 substrate that releases the fluorescent methyl coumarin for 4h. The NO donors were added at 2 mM GSNO, 2 mM DETA-NO, and 0.2 mM $ONOO^-$ (peroxynitrite). The ACE2 inhibitor MLN-4760 was used at 10 µM. A condition was included with Mca alone (without rhACE2) as a negative control of the enzymatic reaction. The figure shows a representative experiment. **(B)** The fluorescence of the cleaved Mca peptide after 90 min of each treatment is shown. **(C)** The enzymatic velocity of ACE2 between minute 0 and 90 from the start of the reaction is shown. Results show the mean ± S.D. of four distinct experiments. B, C; ****p<0.0001 *vs*. Mca.

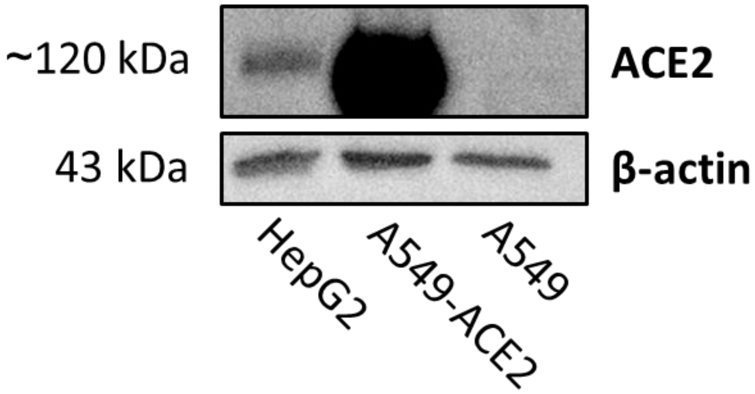

Supplementary figure S2. A549-ACE2 and HepG2 cells express ACE2 protein. Western Blot image showing a clear band that corresponds with ACE2 in HepG2 and A549-ACE2 cells. No band was observed in non-transfected A549 cells. β-actin was used as a loading control.

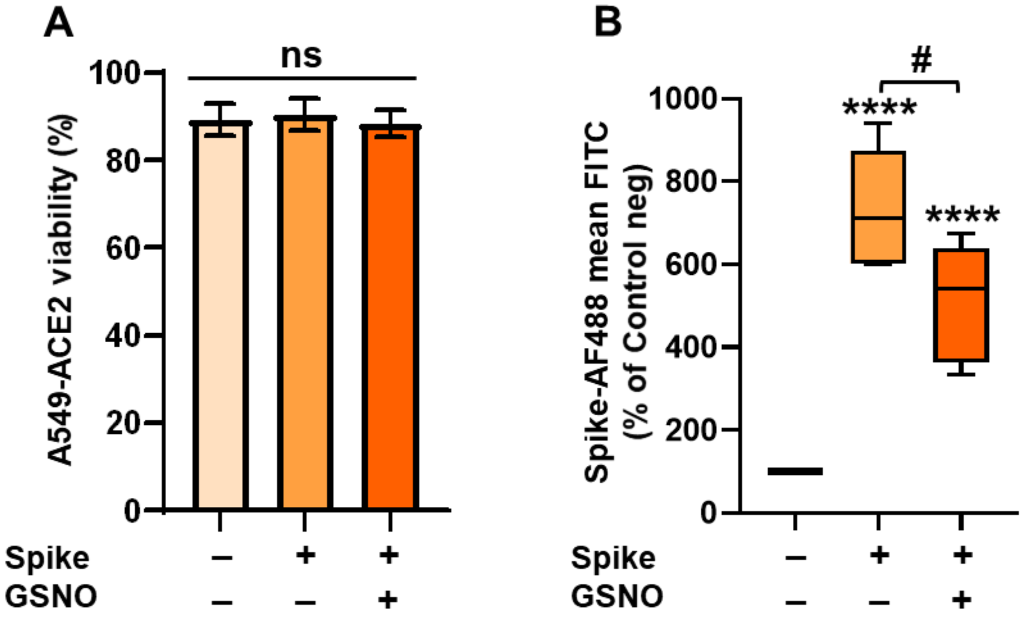

Supplementary figure S3. GSNO inhibits the binding of recombinant gamma variant of SARS-CoV-2 spike to A549-ACE2 cells. (A) Cells ($5x10^5$) were incubated with 2 mM GSNO for 15 min, followed by incubation with the indicated concentrations of spike and GSNO for another 45 min. The viability was determined with DAPI staining. **(B)** The binding of the gamma variant of the spike to A549-ACE2 cells was determined by flow cytometry. Results show the means ± S.D. from 5 different assays. ****$p<0.0001$ *vs*. the untreated control condition; #$p<0.05$ *vs.* the spike condition. **ns:** not significant.

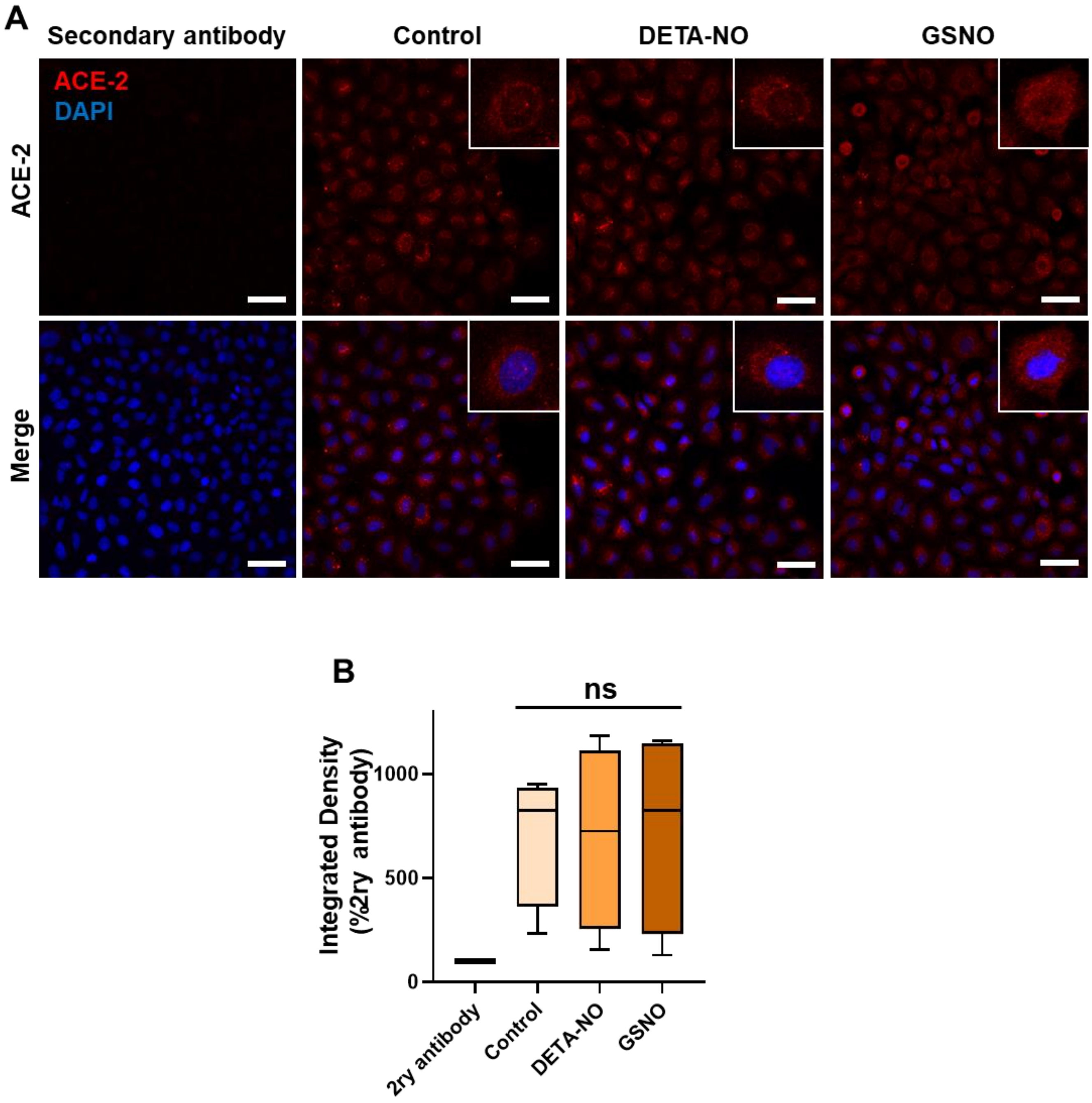

Supplementary figure S4. ACE2 expression and distribution remains unchanged after DETA-NO or GSNO treatment. (A) Representative immunofluorescence images of A549-ACE2 cells treated with DETA-NO or GSNO and stained for ACE2 (red). DAPI (blue) was used to stain nuclei. **(B)** Quantification of the integrated density of the ACE2 signal. Results show the means ± S.D. from 3 different assays. **ns:** not significant. Objective lens: 20X. The scale bar corresponds to 50 µm.
